# *Drosophilamelanogaster* as a model for basal body research

**DOI:** 10.1186/s13630-016-0041-5

**Published:** 2016-07-05

**Authors:** Swadhin Chandra Jana, Mónica Bettencourt-Dias, Bénédicte Durand, Timothy L. Megraw

**Affiliations:** Instituto Gulbenkian de Ciência, Rua da Quinta Grande, número 6, 2780-156 Oeiras, Portugal; Institut NeuroMyogène, CNRS UMR-5310 INSERM-U1217, Université Claude Bernard Lyon-1, Lyon, Villeurbanne, France; Department of Biomedical Sciences, Florida State University, Tallahassee, FL 32306 USA

**Keywords:** Insects, *Drosophila*, Sensory function, Centriole, Male fertility, Motile and immotile cilia, Diverse basal bodies, Evolutionary cell biology

## Abstract

The fruit fly, *Drosophila melanogaster,* is one of the most extensively studied organisms in biological research and has centrioles/basal bodies and cilia that can be modelled to investigate their functions in animals generally. Centrioles are nine-fold symmetrical microtubule-based cylindrical structures required to form centrosomes and also to nucleate the formation of cilia and flagella. When they function to template cilia, centrioles transition into basal bodies. The fruit fly has various types of basal bodies and cilia, which are needed for sensory neuron and sperm function. Genetics, cell biology and behaviour studies in the fruit fly have unveiled new basal body components and revealed different modes of assembly and functions of basal bodies that are conserved in many other organisms, including human, green algae and plasmodium. Here we describe the various basal bodies of *Drosophila*, what is known about their composition, structure and function.

## The fly and its phylogeny

**Fig. 1:**
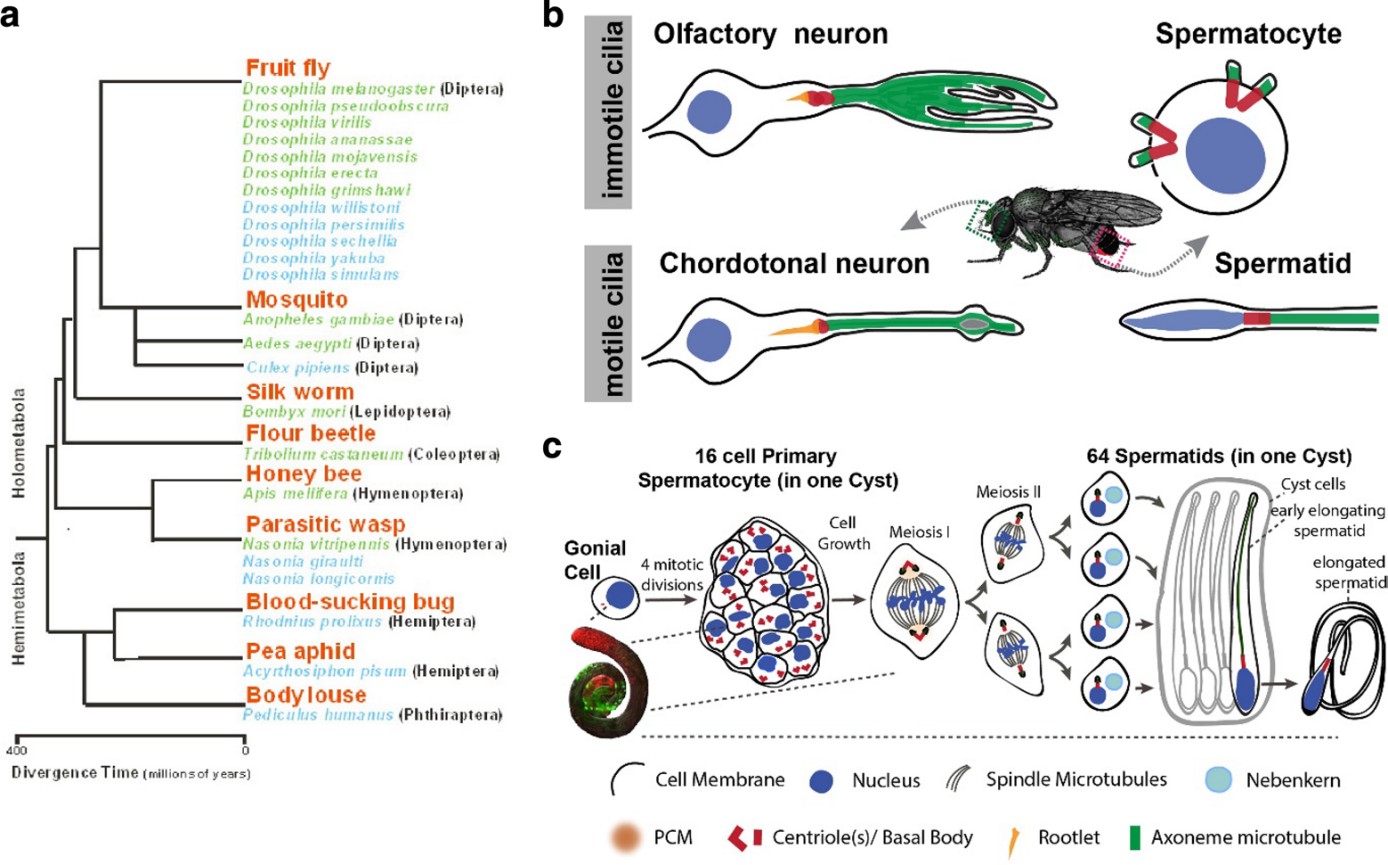
The fruit fly as a cell and evolutionary biology model organism to study basal bodies. **a** Phylogenetic relationships of the insects whose genomes have been sequenced. *Green* indicates genomes that have been fully sequenced (more than 8× coverage), *blue* indicates genomes, where the sequencing has not been completed (less than 8× coverage). The sequenced genomes cover about 350 million years of insect evolution. From: http://www2.bio.ku.dk/insect_genomics/project/. **b** Diagrams, not to scale, of a variety of ciliated cells that grow morphologically different cilia in the adult fly. **c** Schematic representation of *Drosophila* spermatogenesis. A germline stem cell after division gives rise to a gonial cell that in turn undergoes four rounds of incomplete mitotic divisions to produce a 16-cell cyst of interconnected primary spermatocytes. Primary spermatocytes go through a long G2 phase when centrioles/basal bodies elongate and migrate to the cell membrane where each centriole grows a cilium. Each spermatocyte then undergoes two consecutive meiotic divisions without either DNA replication or basal body duplication. As a result, each early spermatid harbours one basal body that templates the sperm flagellum axoneme

The fruit fly *Drosophila melanogaster* is a widely used model organism for biological research in the disciplines of genetics, molecular biology, developmental biology, cell biology and behaviour. Thomas Hunt Morgan initiated the use of *D. melanogaster* with his first studies on heredity at Columbia University published in 1910. The fruit fly offers several advantages for biological studies, including short-generation time (10 days at 25 °C), high fecundity, overall low maintenance costs and relative ease to perform genetics and cell biology experiments. Moreover, about 75 % of known human disease genes have a recognizable match in the fruit fly genome; as such, *Drosophila* is used to understand the molecular mechanisms of diverse human diseases and conditions including cancer, ageing, infertility, neurodegenerative disorders and drug abuse [1]. Finally, the genomes of *D. melanogaster* and eleven other *Drosophila* species have been sequenced and annotated, as well as the genomes of other insects important in human disease, agriculture and manufacturing (e.g. mosquito, silkworm and honeybee) (Fig. 1a). These tools allow biological processes to be studied and compared in evolutionarily related (e.g. *Drosophila* Sp.) [2], close (e.g. mosquito and honeybee) [3] and distant species (e.g. human and plasmodium) [4, 5].

The fruit fly is also a preferred model organism to study centrosome and cilia biology. First, most *Drosophila* proteins required for centrosome and cilia biogenesis are conserved among eukaryotes and are involved in human centrosome and ciliary diseases, such as microcephalies and ciliopathies [5–10]. Second, fruit fly mutants of centrosome and ciliary proteins are not embryonic lethal and can thus be more easily studied for sensory neuron and sperm functions [11, 12]. Third, *Drosophila* harbours diverse basal bodies and cilia that are assembled in different modes that are conserved in many other organisms (Fig. 1b; [5]). Finally, many tools are available to study basal bodies and cilia, such as mutants, RNAi lines, transgenic lines with tagged proteins and antibody reagents [5].

### Diverse cilia in *Drosophila*

While most cells in the fruit fly have no cilia, its type-I sensory neurons and sperm cells have cilia with a variety of configurations and defects in cilia affect diverse sensory functions, such as touch, coordination, taste, olfaction and hearing, and cause sterility [12–14], offering diverse opportunities for cilia and basal body research. Ciliary functions can be tested in *Drosophila* by measuring the response to sensory stimuli, behaviour and/or fertility [12–14].

Sensory reception is mediated by a single cilium on each type-I sensory neuron of the peripheral nervous system (Fig. 1b). Type-I sensory neuron cilia can generally be divided into two categories: (1) cilia in external sensory neurons (9 + 0 type axonemes without dynein arms) are considered immotile [14] and (2) cilia in chordotonal neurons (9 + 0 type axonemes with dynein arms) are believed to be motile [15]. Notably, all cilia on sensory neurons require intraflagellar transport (IFT) for their assembly [16, 17] and the function of olfactory cilia in external sensory neurons require hedgehog signalling, a pathway that is conserved in mammalian cilia [18].

*Drosophila* testes harbour sperm cells and their precursors that also grow cilia (Fig. 1b). While sperm cilia are motile (9 + 2), sperm precursor cells (spermatocytes) have immotile cilia (9 + 0/1) [19–22]. Each spermatocyte has four long centrioles, which convert into basal bodies and therefore assemble four cilia. Following two rounds of meiotic division, spermatids inherit a single basal body that assembles the flagellum (Fig. 1c). The cilia in sperm and sperm precursor cells assemble in an IFT-independent manner [16, 17].

### Centriole identity and structure

**Fig. 2:**
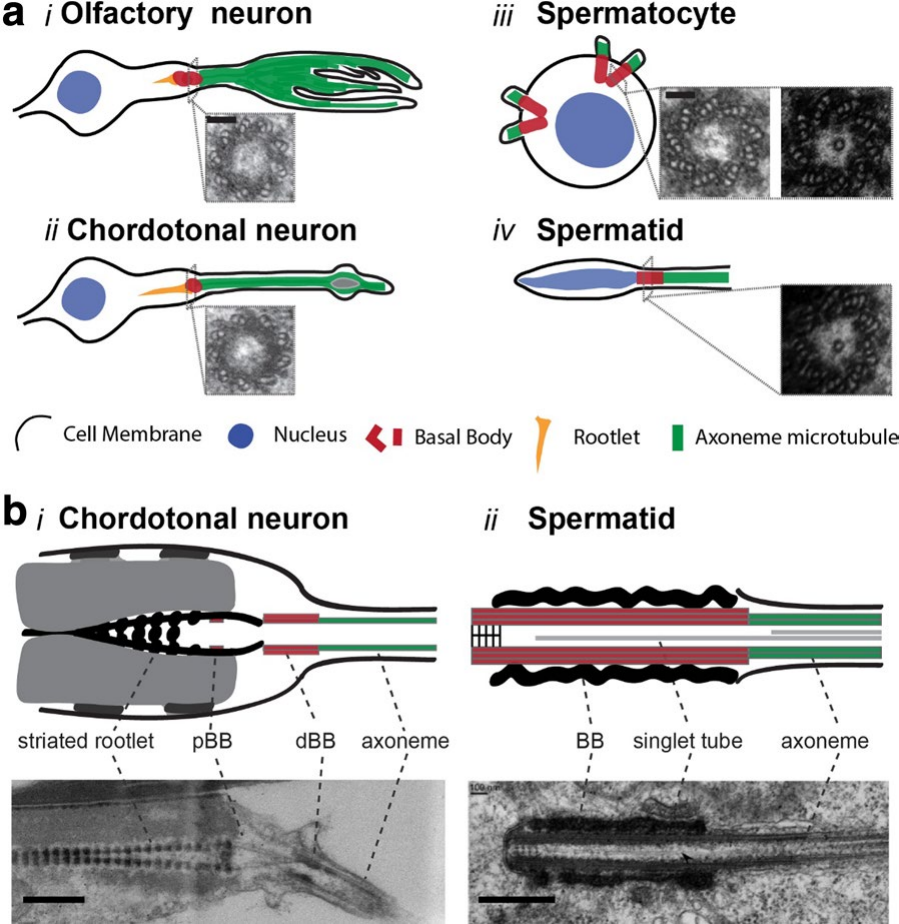
The variety of basal bodies found in *Drosophila*. **a** Representative electron micrographs of the cross section view of the basal body in olfactory neurons (*i*), chordotonal neurons (*ii*), spermatocyte (*iii*) and spermatid (*iv*). **b** Schematics and representative electron micrographs of the longitudinal view of the basal body in chordotonal neurons (*i*) and spermatid (*ii*). BB, pBB and dBB represent basal body, proximal basal body and distal basal body, respectively. *Scale bars* in **a** and **b** represent 100 and 500 nm, respectively. The electron micrographs in **a** are reproduced with permission from [20, 23, 54] and in **b**-*ii* from [20]

Most cycling cells have one centrosome with two centrioles at the beginning of the cell cycle, and two centrosomes, each with two centrioles, after their duplication in the later phases of the cycle (reviewed in [8]). Centrioles within centrosomes and/or basal bodies vary in their length and the organization of the outer microtubules (MT). For example, centrioles/basal bodies in the embryo and sensory neurons are short and made of nine doublet MTs (Fig. 2a i–ii, b-i [14, 23, 24]), whereas those in sperm cells are uniquely long and consist of nine triplet MTs (Fig. 2a iii–iv, b-ii [20, 21]). Thus, flies have a diverse makeup to their centriolar microtubules, with some having doublet MTs, while others have triplet MTs similar to many protists and metazoa, such as plasmodium and mammals [4, 5].

Several EM studies elucidated the structures of *Drosophila* centrioles in cell culture [25], embryos [26], sensory neurons [23] and testis [22, 27]. *Drosophila* centrioles do not have distinct distal or subdistal appendages as their mammalian counterparts, and mother and daughter centrioles are indistinguishable at the EM level except for by their relative juxtaposition (the daughter attached to the mother at the proximal base) [28]. Curiously, despite lacking the distal and subdistal appendages on mother centrioles, *Drosophila* do have orthologs of key protein components of these structures such as Cep164 (CG9170) [10] and ninein (Bsg25D) [29]. Moreover, proteins have been identified that are specific for daughter centrioles like centrobin [30], and transgenes expressing the PACT domain from pericentrin-like protein (Plp) are enriched at the mother centriole [23, 31, 32]. In ciliated chordotonal neurons, these markers indicate that the cilium grows from the mother centriole. Thus, however, the lack of overt distal structures that adorn mother centrioles and are required in other organisms for ciliogenesis, mother centrioles are nevertheless distinguished by their ability to form cilia in *Drosophila*. Functionally, centrobin appears to confer daughter identity, as it restricts the daughter centriole from engaging in cilium assembly [23].

## Basal body origins and structure

### Centriole to basal body conversion

*Drosophila* basal bodies, which display many unique features that are conserved in many other organisms, are converted from canonically formed centrioles in all ciliated tissues. In sensory neurons, no direct observation of the conversion of centrioles to basal bodies has been published. However, serial sections of neuronal cells by EM show centriolar structures only at the base of the cilia [33] and centriolar proteins only label the ciliary base of sensory neurons by microscopic imaging [23, 34–37]. Based on data from other arthropod chordotonal cilia, we can expect thin fibrous structures linking the MTs at the distal centriole to the membrane connections in the neurons [38], but complete description of how basal bodies anchor to membranes in *Drosophila* ciliated neurons is pending.

The centriole to basal body conversion was documented in sperm cells by exhaustive electron microscopy observations ([22] and recently [20, 21]) and can be followed by live imaging of centriole behaviour during differentiation of sperm cells [39]. The basal bodies in the *Drosophila* testis grow exceptionally long during spermatocyte maturation (Fig. 1c) [22, 27, 40]. These giant centrioles/basal bodies are about 1.3 µm long, including the short cilium-like region at their distal end, which is approximately 400 nm long and is the precursor for formation of the long sperm flagellum [41]. The basal bodies and short cilia in spermatocytes are unusual in several respects: the cilia assemble in G2 phase, all four basal bodies anchor at the plasma membrane and assemble cilia, and the cilia persist through two meiotic cell divisions (Fig. 1c) [21, 22, 27]. Inside the lumen of the spermatocyte and spermatid basal body, there is a single central tubule that is variable in length, but can extend into the transition zone and coincide with the axonemal central pair (Fig. 2a, b) [19, 20, 42]. This single MT appears to be stabilized by Bld10, a MT-binding protein required for centriole elongation and stability in the fruit fly, and promotes the formation and/or stability of the central pair of MTs within the sperm axoneme [20]. Despite the lack of distal appendages, spermatocyte and spermatid basal bodies have thin fibrous structures that link the C tubules at the distal centriole to the membrane.

In the early spermatid, the basal body migrates to the nucleus and anchors to the nuclear envelope. As spermiogenesis proceeds, a pericentriolar material (PCM)-like toroid structure called the “centriolar adjunct” forms, encircling the proximal base of the giant centriole [43]. The function of the centriolar adjunct is unclear, but it appears to nurture the assembly of a new centriole during spermatozoan formation. Within the centriolar adjunct a unique structure forms called the proximal centriole-like structure (PCL), which contains several centriole proteins including Ana1, Ana2, Bld10, Sas-4 and Sas-6 [42, 44, 45]. Assembly of the PCL requires the centriole biogenesis proteins Sas-6 and Sak/PLK4, and has a unique requirement for Poc1 that is not required for centriole assembly generally in *Drosophila* [44]. The PCL appears during spermatid differentiation and appears to be an atypical procentriole, which forms within the centriolar adjunct and might be reduced later on [46]. When delivered to the embryo at fertilization along with the giant basal body, the remainder of the PCL matures into a centriole, duplicates and assembles a centrosome that contributes to the first mitosis of the embryo [45].

The spermatozoan axoneme grows to a length of approximately 1800 µm—this is very long compared to humans for example, where the sperm tail is about 50 µm long. As the axoneme assembles in the spermatid, it appears exposed in the cytoplasm. However, the distal ~2 µm of the axoneme is encased in membrane that is contiguous with the plasma membrane but is anchored to the axoneme at a structure called the “ring centriole” [40, 47, 48]. This distal portion of the growing flagellum appears to be a cilium with a distinct compartment, with typical transition zone proteins like unc, Cby, Mks1 and Cep290 localized at the ring centriole at the cilium base, despite the absence of a basal body [34, 49–51]. Thus, there is no basal body structure at the base of the spermatid distal compartmentalized cilium. The axoneme extends through the cytoplasm to the basal body anchored at the nucleus, yet the ring centriole appears to form a membrane barrier, which, as the axoneme grows, behaves as a migrating ciliary gate [51]. In the mouse, spermatozoan development follows a similar path, where a structure called the annulus appears to be analogous to the ring centriole [51].

The sensory neurons in *Drosophila* harbour ciliary rootlets of variable lengths depending on the neuron type (Fig. 2b-i), but these structures are not found in the testis [22, 36]. The ciliary rootlet, a cytoskeletal structure comprised of striated fibres, assembles at the basal body in many ciliated organisms and cell types including insects and human [38]. Rootletin is a major component of rootlets in *Drosophila* and is required for rootlet assembly, but not for cilium assembly, and rootlets are necessary for sensory neuron function [36, 52].

## Basal body life cycle and other functions

### Does the basal body also have the function of a centrosome?

Sensory neurons are terminally differentiated cells with the centriole pair residing at the tip of a single dendrite where one assembles a cilium. The basal bodies do not appear to function as an active MT-organizing centre (MTOC). In spermatocytes, in G2 phase, all 4 duplicated centrioles convert to basal bodies, dock to the plasma membrane and each one grows a primary cilium-like structure [20–22, 53, 54]. These cilia-like structures are not disassembled during meiosis. Basal bodies, together with the cilia-like structures, are internalized and mature into centrosomes that organize the meiotic spindle. Hence, basal bodies are able to simultaneously organize cilia and spindle poles [22] during *Drosophila* male meiosis (Fig. 1c). In mouse neuronal stem cells, a somewhat similar process occurs: the primary cilium is incompletely resorbed and the basal body with residual cilium participates in the following asymmetric mitosis [55].

### Do *Drosophila* have basal bodies at all stages of their life cycle? If not when?

Ciliated cells are present only as type-I sensory neurons, which develop during mid-embryogenesis, and in spermatogenic cells at the beginning of larval stages in *Drosophila*. Ciliated neurons in adults are built during metamorphosis from sensory precursors derived from larval imaginal discs. Basal bodies are required to build the sensory cilia [11] and are maintained during ageing of sensory cells [36, 52]. In male germ cells, basal bodies are formed in spermatocytes and maintained during spermatid maturation. In mature sperm, basal bodies are still present as seen by EM [22] but several basal body/centriolar markers are reduced [42, 44, 56, 57], illustrating the remodelling of the basal body that occurs in late spermiogenesis and also observed in several other animal species by a phenomenon called “centrosome reduction” (see [58, 59]).

### Identification of basal body components

There have been no proteomics performed on isolated *Drosophila* basal bodies, but there was a proteomics survey done on isolated mature sperm [60]. The spermatozoan typically undergoes centrosome reduction during spermatogenesis [58, 61]. So while this study did not reveal any new basal body components, it did reveal centrosome and centriole proteins that were retained in the mature sperm (see Table 1) [60]. Since *Drosophila* sperm require functional flagella, and flies have somatic cilia only on sensory neurons where they are required for a variety of sensory functions, genetic screens that involved neurological motor activity or male fertility identified some cilium and basal body components. Table 1 summarizes genetic, RNAi, and proteomic screens that identified centriole components.

**Table 1:** Proteomic, RNAi and genomic screens that identified *Drosophila* centriole or centrosome proteins

Type of screen	System	Proteins identified	References
Genetic screen for mechanosensation defects	In vivo genetic screen	Unc, Asterless (MecD), Cep290 (MecH)	[13, 62, 63]
Genetic screen for male infertility	In vivo genetic screen	Asterless, Spd-2	[64, 65]
RNAi	Cell culture	Ana1, Ana2, Ana3	[66]
RNAi	Cell culture	Bld10, CP110, Cep97, Rcd4	[67]
Proteomic	Mature sperm	Ana1, Ana3, Asp, Bld10, Grip163, Ninein, Plp, Rootletin	[60]
Proteomic	Isolated blastoderm embryo centrosomes	CG11148, Cort, Crm, eIF-4a, Feo, Lam, Nup153, TFAM	[68]
Proteomic	Isolated blastoderm embryo centrosomes	Ote; new phosphorylation sites mapped in known centrosome proteins	[69]

### Summary of notable basal body findings

To summarize, *Drosophila* harbour diverse centriole/basal bodies with doublet and triplet MTs. A notable feature associated with basal bodies in *Drosophila* is a lack of distal or subdistal appendages. A unique feature at the sperm basal body is the PCL: a procentriole that appears in the differentiating spermatid within a PCM-like structure called the centriolar adjunct. Another notable feature in the *Drosophila* testis is the ring centriole. The ring centriole is a unique example of a transition zone-like structure that creates a cilium compartment without a canonical basal body. A fourth notable feature, residing in the centre of the long spermatocyte and spermatid basal body, is a clear central tubule, which is probably a dynamic MT. It extends from the hub of the cartwheel at the proximal end of the basal body to the distal end, where it transitions into the central pair of MTs in the axoneme. Finally, another notable feature associated with the neuronal basal body is the rootlet, a conserved cytoskeletal structure comprised striated fibres. Rootletin, a conserved component of root-like structures, is required for rootlet assembly and thereby supports sensory cilia functions.

### Strengths and future of basal body research in *Drosophila*

Unique advantages offered by *D. melanogaster* as a model for basal body research is the variety of basal bodies encountered in this organism that are also found in many eukaryotes, as well as limited requirements for cilia in this organism to sensory neurons and sperm cells. The absence of basal bodies or disruption of basal body proteins in *Drosophila* results in the loss of sensory functions (touch, hearing, olfaction and taste perceptions) and male fertility. Genetic screens are therefore possible to identify the components involved in the above functions. *Drosophila* is also a great model to study alternative modes of: cilia assembly (IFT-independent in sperm); transition zone function (ring centriole; appears conserved in vertebrates); and centriole biogenesis (the PCL). *Drosophila* is also an important model to study conventional modes of: cilia assembly (IFT-dependent in neurons); centriole biogenesis and elongation (the centrioles of different types of MTs and lengths in neurons and sperm cells); and ciliary rootlet biogenesis (the rootlet in neurons). Moreover, the recent sequencing of the genomes of several other *Drosophila* species and other insects permits the applications of comparative studies of basal body assembly and function.

## Abbreviations

MT: microtubules
MTOC: microtubule-organizing center
IFT: intraflagellar transport
PCM: pericentriolar material
PCL: procentriole-like structure
BB: basal body
pBB: proximal basal body
dBB: distal basal body
